# 5-Meth­oxy-2-[(2-morpholinoeth­yl)­iminio­meth­yl]phenolate

**DOI:** 10.1107/S1600536809013786

**Published:** 2009-04-18

**Authors:** Nooraziah Mohd Lair, Hapipah Mohd Ali, Seik Weng Ng

**Affiliations:** a Department of Chemistry, University of Malaya, 50603 Kuala Lumpur, Malaysia

## Abstract

Each of the two independent mol­ecules of the title comound, C_14_H_20_N_2_O_3_, exists in the zwitterionic form as the imino N atoms are protonated. The =N—H unit forms an intra­molecular hydrogen bond to the negatively charged O atom, and also a weaker intermolecular N—H⋯O bond, the latter resulting in inversion dimers.

## Related literature

For the structure of 2-[(2-morpholinoethyl­imino)­methyl]­phenol, see: Petek *et al.* (2005 ▶).
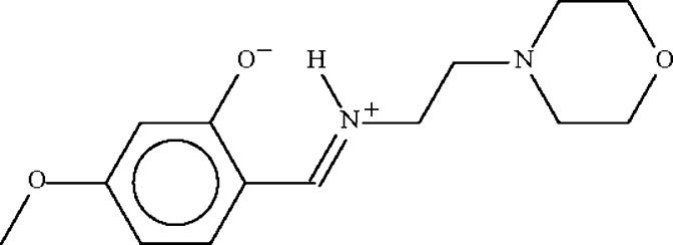


## Experimental

### 

#### Crystal data

C_14_H_20_N_2_O_3_
*M* *_r_* = 264.32
Triclinic, 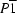

*a* = 10.4022 (2) Å
*b* = 10.7340 (2) Å
*c* = 14.3497 (3) Å
α = 83.523 (1)°
β = 74.810 (1)°
γ = 60.768 (1)°
*V* = 1349.13 (5) Å^3^
*Z* = 4
Mo *K*α radiation
μ = 0.09 mm^−1^
*T* = 100 K
0.25 × 0.25 × 0.25 mm

#### Data collection

Bruker SMART APEX diffractometer
Absorption correction: none
9402 measured reflections
5948 independent reflections
4990 reflections with *I* > 2σ(*I*)
*R*_int_ = 0.019

#### Refinement

*R*[*F* ^2^ > 2σ(*F* ^2^)] = 0.040
*wR*(*F* ^2^) = 0.122
*S* = 1.04
5948 reflections
353 parameters
2 restraints
H atoms treated by a mixture of independent and constrained refinement
Δρ_max_ = 0.31 e Å^−3^
Δρ_min_ = −0.28 e Å^−3^

### 

Data collection: *APEX2* (Bruker, 2008 ▶); cell refinement: *SAINT* (Bruker, 2008 ▶); data reduction: *SAINT*; program(s) used to solve structure: *SHELXS97* (Sheldrick, 2008 ▶); program(s) used to refine structure: *SHELXL97* (Sheldrick, 2008 ▶); molecular graphics: *X-SEED* (Barbour, 2001 ▶); software used to prepare material for publication: *publCIF* (Westrip, 2009 ▶).

## Supplementary Material

Crystal structure: contains datablocks global, I. DOI: 10.1107/S1600536809013786/tk2422sup1.cif

Structure factors: contains datablocks I. DOI: 10.1107/S1600536809013786/tk2422Isup2.hkl

Additional supplementary materials:  crystallographic information; 3D view; checkCIF report

## Figures and Tables

**Table 1:** Hydrogen-bond geometry (Å, °)

*D*—H⋯*A*	*D*—H	H⋯*A*	*D*⋯*A*	*D*—H⋯*A*
N1—H1⋯O1	0.88 (1)	1.96 (1)	2.6489 (15)	133 (2)
N1—H1⋯O1^i^	0.88 (1)	2.32 (1)	2.9570 (18)	129 (1)
N3—H3⋯O4	0.89 (1)	2.02 (1)	2.6930 (15)	132 (1)
N3—H3⋯O4^ii^	0.89 (1)	2.29 (1)	2.9505 (18)	132 (1)

Symmetry codes: (i) 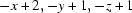; (ii) 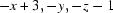.

**Fig. 1.**
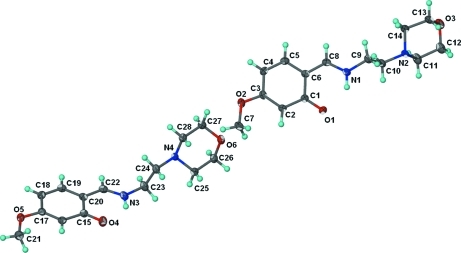
Thermal ellipsoid plot (Barbour, 2001) of the two independent molecules of C14H20N2O3 at the 70% probability level. H atoms are drawn as spheres of arbitrary radius.

## supplementary crystallographic information

### Experimental

2-Hydroxy-4-methoxybenzaldehyde (0.3 g, 2 mmol) and *N*-(2-aminoethyl)morpholine (0.26 g, 2 mmol) were heated in acidified ethanol (50 ml) for 8 h. The solvent was removed to give an oil; crystals appeared in the oil after several days.

### Refinement

H atoms were placed at calculated positions (C—H = 0.95–0.99 Å) and were treated as riding on their parent C atoms, with *U*(H) set to 1.2–1.5 times *U*_eq_(C). The iminium H atoms were located in a difference Fourier map, and were refined with a distance restraint of N—H = 0.88 (1) Å; their temperature factors were refined.

### Crystal data

**Table:**

C_14_H_20_N_2_O_3_	*Z* = 4
*M**_r_* = 264.32	*F*(000) = 568
Triclinic, *P*1	*D*_x_ = 1.301 Mg m^−^^3^
Hall symbol: -P 1	Mo *K*α radiation, λ = 0.71073 Å
*a* = 10.4022 (2) Å	Cell parameters from 4712 reflections
*b* = 10.7340 (2) Å	θ = 2.3–28.3°
*c* = 14.3497 (3) Å	µ = 0.09 mm^−^^1^
α = 83.523 (1)°	*T* = 100 K
β = 74.810 (1)°	Block, yellow
γ = 60.768 (1)°	0.25 × 0.25 × 0.25 mm
*V* = 1349.13 (5) Å^3^	

### Data collection

**Table:**

Bruker SMART APEX diffractometer	4990 reflections with *I* > 2σ(*I*)
Radiation source: fine-focus sealed tube	*R*_int_ = 0.019
graphite	θ_max_ = 27.5°, θ_min_ = 1.5°
ω scans	*h* = −13→13
9402 measured reflections	*k* = −13→13
5948 independent reflections	*l* = −18→16

### Refinement

**Table:**

Refinement on *F*^2^	Primary atom site location: structure-invariant direct methods
Least-squares matrix: full	Secondary atom site location: difference Fourier map
*R*[*F*^2^ > 2σ(*F*^2^)] = 0.040	Hydrogen site location: inferred from neighbouring sites
*wR*(*F*^2^) = 0.122	H atoms treated by a mixture of independent and constrained refinement
*S* = 1.04	*w* = 1/[σ^2^(*F*_o_^2^) + (0.0696*P*)^2^ + 0.3249*P*] where *P* = (*F*_o_^2^ + 2*F*_c_^2^)/3
5948 reflections	(Δ/σ)_max_ = 0.001
353 parameters	Δρ_max_ = 0.31 e Å^−^^3^
2 restraints	Δρ_min_ = −0.28 e Å^−^^3^

### Fractional atomic coordinates and isotropic or equivalent isotropic displacement parameters (Å^2^)

**Table:**

	*x*	*y*	*z*	*U*_iso_*/*U*_eq_	
O1	0.98316 (10)	0.43387 (9)	0.41326 (7)	0.0206 (2)	
O2	0.96310 (10)	0.15768 (9)	0.18272 (6)	0.0212 (2)	
O3	0.38917 (12)	0.92759 (12)	0.95202 (8)	0.0352 (3)	
O4	1.50070 (10)	−0.11554 (10)	−0.55972 (7)	0.0248 (2)	
O5	1.47841 (10)	−0.36606 (9)	−0.80663 (6)	0.0207 (2)	
O6	0.93660 (11)	0.37780 (10)	−0.02785 (6)	0.0226 (2)	
N1	0.77639 (12)	0.49804 (11)	0.57933 (8)	0.0179 (2)	
H1	0.8542 (15)	0.5066 (19)	0.5426 (11)	0.039 (5)*	
N2	0.60959 (11)	0.75546 (11)	0.78788 (7)	0.0168 (2)	
N3	1.23596 (12)	0.09777 (11)	−0.46514 (8)	0.0201 (2)	
H3	1.3361 (10)	0.0512 (16)	−0.4734 (12)	0.034 (5)*	
N4	1.02064 (11)	0.24956 (10)	−0.21452 (7)	0.0163 (2)	
C1	0.91918 (13)	0.37426 (12)	0.38550 (9)	0.0157 (2)	
C2	0.98177 (13)	0.29765 (12)	0.29480 (9)	0.0165 (2)	
H2	1.0732	0.2909	0.2540	0.020*	
C3	0.91073 (14)	0.23419 (12)	0.26664 (9)	0.0165 (2)	
C4	0.77322 (14)	0.24178 (12)	0.32475 (9)	0.0178 (3)	
H4	0.7249	0.1988	0.3032	0.021*	
C5	0.71188 (13)	0.31159 (12)	0.41178 (9)	0.0174 (2)	
H5	0.6208	0.3158	0.4512	0.021*	
C6	0.78113 (13)	0.37826 (12)	0.44499 (9)	0.0157 (2)	
C7	1.11022 (15)	0.12990 (14)	0.12544 (9)	0.0226 (3)	
H7A	1.1382	0.0687	0.0699	0.034*	
H7B	1.1850	0.0817	0.1647	0.034*	
H7C	1.1080	0.2203	0.1026	0.034*	
C8	0.72030 (14)	0.43854 (12)	0.53895 (9)	0.0171 (2)	
H8	0.6315	0.4357	0.5759	0.021*	
C9	0.72088 (15)	0.54438 (13)	0.68036 (9)	0.0193 (3)	
H9A	0.8066	0.5011	0.7122	0.023*	
H9B	0.6470	0.5115	0.7139	0.023*	
C10	0.64529 (14)	0.70646 (13)	0.68841 (9)	0.0180 (3)	
H10A	0.7136	0.7401	0.6460	0.022*	
H10B	0.5506	0.7489	0.6657	0.022*	
C11	0.54085 (16)	0.91155 (14)	0.78853 (10)	0.0248 (3)	
H11A	0.4522	0.9524	0.7595	0.030*	
H11B	0.6154	0.9399	0.7486	0.030*	
C12	0.49055 (17)	0.97165 (15)	0.88980 (10)	0.0297 (3)	
H12A	0.5807	0.9394	0.9161	0.036*	
H12B	0.4397	1.0772	0.8877	0.036*	
C13	0.46395 (18)	0.77580 (18)	0.95435 (10)	0.0333 (4)	
H13A	0.3965	0.7439	0.9998	0.040*	
H13B	0.5568	0.7415	0.9780	0.040*	
C14	0.50611 (16)	0.71168 (16)	0.85489 (10)	0.0271 (3)	
H14A	0.5558	0.6062	0.8584	0.032*	
H14B	0.4134	0.7446	0.8313	0.032*	
C15	1.42646 (14)	−0.13723 (13)	−0.60736 (9)	0.0178 (3)	
C16	1.49985 (14)	−0.24612 (12)	−0.68219 (9)	0.0176 (3)	
H16	1.6069	−0.3044	−0.6959	0.021*	
C17	1.41790 (14)	−0.26689 (12)	−0.73376 (9)	0.0168 (2)	
C18	1.25664 (14)	−0.18526 (13)	−0.71453 (9)	0.0187 (3)	
H18	1.2014	−0.2031	−0.7497	0.022*	
C19	1.18346 (14)	−0.08089 (13)	−0.64448 (9)	0.0184 (3)	
H19	1.0762	−0.0254	−0.6318	0.022*	
C20	1.26264 (14)	−0.05270 (12)	−0.59010 (9)	0.0172 (2)	
C21	1.64017 (15)	−0.44652 (14)	−0.83657 (10)	0.0248 (3)	
H21A	1.6685	−0.5143	−0.8881	0.037*	
H21B	1.6807	−0.4988	−0.7815	0.037*	
H21C	1.6821	−0.3815	−0.8606	0.037*	
C22	1.17933 (14)	0.05782 (13)	−0.51997 (9)	0.0185 (3)	
H22	1.0723	0.1080	−0.5116	0.022*	
C23	1.14126 (15)	0.21047 (13)	−0.39115 (9)	0.0216 (3)	
H23A	1.0390	0.2691	−0.4037	0.026*	
H23B	1.1866	0.2735	−0.3935	0.026*	
C24	1.12827 (14)	0.14453 (12)	−0.29159 (9)	0.0181 (3)	
H24A	1.0959	0.0721	−0.2928	0.022*	
H24B	1.2295	0.0951	−0.2769	0.022*	
C25	1.07626 (14)	0.34487 (13)	−0.19663 (9)	0.0192 (3)	
H25A	1.0905	0.3981	−0.2560	0.023*	
H25B	1.1758	0.2874	−0.1802	0.023*	
C26	0.96492 (15)	0.44957 (13)	−0.11458 (9)	0.0208 (3)	
H26A	1.0061	0.5105	−0.1025	0.025*	
H26B	0.8682	0.5123	−0.1335	0.025*	
C27	0.88279 (15)	0.28513 (14)	−0.04521 (9)	0.0222 (3)	
H27A	0.7851	0.3425	−0.0640	0.027*	
H27B	0.8647	0.2350	0.0150	0.027*	
C28	0.99553 (15)	0.17629 (13)	−0.12430 (9)	0.0197 (3)	
H28A	1.0928	0.1171	−0.1052	0.024*	
H28B	0.9562	0.1126	−0.1341	0.024*	

### Atomic displacement parameters (Å^2^)

**Table:**

	*U*^11^	*U*^22^	*U*^33^	*U*^12^	*U*^13^	*U*^23^
O1	0.0209 (5)	0.0253 (5)	0.0202 (5)	−0.0151 (4)	−0.0019 (4)	−0.0037 (4)
O2	0.0215 (5)	0.0235 (4)	0.0180 (5)	−0.0106 (4)	−0.0018 (4)	−0.0062 (4)
O3	0.0228 (5)	0.0519 (7)	0.0277 (6)	−0.0175 (5)	0.0070 (4)	−0.0198 (5)
O4	0.0196 (5)	0.0286 (5)	0.0258 (5)	−0.0094 (4)	−0.0061 (4)	−0.0070 (4)
O5	0.0196 (5)	0.0214 (4)	0.0209 (5)	−0.0100 (4)	−0.0010 (4)	−0.0069 (4)
O6	0.0290 (5)	0.0254 (5)	0.0166 (5)	−0.0158 (4)	−0.0034 (4)	−0.0028 (4)
N1	0.0177 (5)	0.0197 (5)	0.0152 (5)	−0.0093 (4)	−0.0004 (4)	−0.0020 (4)
N2	0.0167 (5)	0.0195 (5)	0.0132 (5)	−0.0089 (4)	−0.0008 (4)	−0.0013 (4)
N3	0.0181 (5)	0.0199 (5)	0.0174 (5)	−0.0061 (4)	−0.0012 (4)	−0.0033 (4)
N4	0.0173 (5)	0.0173 (5)	0.0141 (5)	−0.0087 (4)	−0.0014 (4)	−0.0028 (4)
C1	0.0158 (6)	0.0148 (5)	0.0162 (6)	−0.0071 (4)	−0.0045 (5)	0.0014 (4)
C2	0.0153 (6)	0.0170 (5)	0.0158 (6)	−0.0074 (5)	−0.0022 (5)	0.0007 (4)
C3	0.0174 (6)	0.0140 (5)	0.0151 (6)	−0.0047 (4)	−0.0048 (5)	−0.0003 (4)
C4	0.0172 (6)	0.0178 (5)	0.0216 (6)	−0.0095 (5)	−0.0074 (5)	0.0008 (5)
C5	0.0142 (5)	0.0172 (5)	0.0194 (6)	−0.0072 (5)	−0.0037 (5)	0.0027 (5)
C6	0.0144 (5)	0.0149 (5)	0.0160 (6)	−0.0060 (4)	−0.0031 (4)	0.0008 (4)
C7	0.0221 (6)	0.0224 (6)	0.0182 (6)	−0.0087 (5)	0.0009 (5)	−0.0033 (5)
C8	0.0146 (6)	0.0164 (5)	0.0180 (6)	−0.0066 (5)	−0.0022 (5)	0.0011 (4)
C9	0.0224 (6)	0.0213 (6)	0.0139 (6)	−0.0115 (5)	−0.0012 (5)	−0.0015 (5)
C10	0.0171 (6)	0.0209 (6)	0.0139 (6)	−0.0079 (5)	−0.0021 (5)	−0.0015 (5)
C11	0.0258 (7)	0.0208 (6)	0.0200 (7)	−0.0048 (5)	−0.0047 (5)	−0.0034 (5)
C12	0.0300 (7)	0.0288 (7)	0.0245 (7)	−0.0109 (6)	0.0001 (6)	−0.0090 (6)
C13	0.0389 (8)	0.0540 (9)	0.0177 (7)	−0.0346 (8)	0.0055 (6)	−0.0074 (6)
C14	0.0290 (7)	0.0415 (8)	0.0183 (7)	−0.0251 (7)	0.0019 (5)	−0.0056 (6)
C15	0.0190 (6)	0.0188 (5)	0.0159 (6)	−0.0099 (5)	−0.0034 (5)	0.0011 (4)
C16	0.0146 (6)	0.0178 (5)	0.0189 (6)	−0.0073 (5)	−0.0021 (5)	−0.0004 (5)
C17	0.0199 (6)	0.0151 (5)	0.0150 (6)	−0.0093 (5)	−0.0014 (5)	0.0001 (4)
C18	0.0187 (6)	0.0213 (6)	0.0191 (6)	−0.0110 (5)	−0.0063 (5)	0.0013 (5)
C19	0.0158 (6)	0.0184 (6)	0.0187 (6)	−0.0071 (5)	−0.0037 (5)	0.0029 (5)
C20	0.0176 (6)	0.0170 (5)	0.0152 (6)	−0.0078 (5)	−0.0026 (5)	0.0013 (4)
C21	0.0208 (6)	0.0220 (6)	0.0275 (7)	−0.0086 (5)	0.0008 (5)	−0.0084 (5)
C22	0.0176 (6)	0.0188 (6)	0.0170 (6)	−0.0080 (5)	−0.0031 (5)	0.0026 (5)
C23	0.0216 (6)	0.0195 (6)	0.0188 (6)	−0.0061 (5)	−0.0023 (5)	−0.0051 (5)
C24	0.0168 (6)	0.0170 (5)	0.0183 (6)	−0.0055 (5)	−0.0044 (5)	−0.0038 (5)
C25	0.0199 (6)	0.0207 (6)	0.0185 (6)	−0.0115 (5)	−0.0021 (5)	−0.0024 (5)
C26	0.0253 (7)	0.0204 (6)	0.0187 (6)	−0.0127 (5)	−0.0035 (5)	−0.0028 (5)
C27	0.0242 (7)	0.0271 (6)	0.0183 (6)	−0.0158 (6)	−0.0016 (5)	−0.0017 (5)
C28	0.0237 (6)	0.0206 (6)	0.0178 (6)	−0.0128 (5)	−0.0048 (5)	0.0000 (5)

### Geometric parameters (Å, °)

**Table:**

O1—C1	1.2741 (15)	C10—H10B	0.9900
O2—C3	1.3652 (14)	C11—C12	1.5115 (18)
O2—C7	1.4342 (15)	C11—H11A	0.9900
O3—C13	1.423 (2)	C11—H11B	0.9900
O3—C12	1.4253 (18)	C12—H12A	0.9900
O4—C15	1.2673 (16)	C12—H12B	0.9900
O5—C17	1.3691 (14)	C13—C14	1.5172 (18)
O5—C21	1.4308 (15)	C13—H13A	0.9900
O6—C27	1.4274 (15)	C13—H13B	0.9900
O6—C26	1.4291 (15)	C14—H14A	0.9900
N1—C8	1.3071 (16)	C14—H14B	0.9900
N1—C9	1.4581 (16)	C15—C16	1.4436 (17)
N1—H1	0.883 (9)	C15—C20	1.4518 (17)
N2—C11	1.4657 (16)	C16—C17	1.3702 (18)
N2—C10	1.4673 (15)	C16—H16	0.9500
N2—C14	1.4676 (16)	C17—C18	1.4263 (17)
N3—C22	1.3097 (17)	C18—C19	1.3657 (17)
N3—C23	1.4625 (15)	C18—H18	0.9500
N3—H3	0.889 (9)	C19—C20	1.4187 (18)
N4—C24	1.4654 (15)	C19—H19	0.9500
N4—C28	1.4685 (16)	C20—C22	1.4068 (17)
N4—C25	1.4706 (15)	C21—H21A	0.9800
C1—C2	1.4383 (16)	C21—H21B	0.9800
C1—C6	1.4486 (16)	C21—H21C	0.9800
C2—C3	1.3724 (17)	C22—H22	0.9500
C2—H2	0.9500	C23—C24	1.5246 (18)
C3—C4	1.4229 (17)	C23—H23A	0.9900
C4—C5	1.3619 (17)	C23—H23B	0.9900
C4—H4	0.9500	C24—H24A	0.9900
C5—C6	1.4207 (17)	C24—H24B	0.9900
C5—H5	0.9500	C25—C26	1.5177 (17)
C6—C8	1.4096 (17)	C25—H25A	0.9900
C7—H7A	0.9800	C25—H25B	0.9900
C7—H7B	0.9800	C26—H26A	0.9900
C7—H7C	0.9800	C26—H26B	0.9900
C8—H8	0.9500	C27—C28	1.5138 (17)
C9—C10	1.5231 (16)	C27—H27A	0.9900
C9—H9A	0.9900	C27—H27B	0.9900
C9—H9B	0.9900	C28—H28A	0.9900
C10—H10A	0.9900	C28—H28B	0.9900
			
C3—O2—C7	117.10 (10)	C14—C13—H13B	109.4
C13—O3—C12	108.59 (10)	H13A—C13—H13B	108.0
C17—O5—C21	117.66 (10)	N2—C14—C13	109.57 (11)
C27—O6—C26	109.87 (9)	N2—C14—H14A	109.8
C8—N1—C9	123.55 (11)	C13—C14—H14A	109.8
C8—N1—H1	116.5 (12)	N2—C14—H14B	109.8
C9—N1—H1	119.9 (12)	C13—C14—H14B	109.8
C11—N2—C10	108.28 (10)	H14A—C14—H14B	108.2
C11—N2—C14	109.75 (10)	O4—C15—C16	122.00 (11)
C10—N2—C14	112.35 (10)	O4—C15—C20	121.65 (11)
C22—N3—C23	122.71 (11)	C16—C15—C20	116.34 (11)
C22—N3—H3	116.9 (11)	C17—C16—C15	121.19 (11)
C23—N3—H3	120.4 (11)	C17—C16—H16	119.4
C24—N4—C28	109.97 (9)	C15—C16—H16	119.4
C24—N4—C25	111.83 (9)	O5—C17—C16	124.99 (11)
C28—N4—C25	108.10 (10)	O5—C17—C18	113.13 (11)
O1—C1—C2	121.77 (11)	C16—C17—C18	121.87 (11)
O1—C1—C6	121.36 (11)	C19—C18—C17	118.51 (11)
C2—C1—C6	116.85 (11)	C19—C18—H18	120.7
C3—C2—C1	120.63 (11)	C17—C18—H18	120.7
C3—C2—H2	119.7	C18—C19—C20	122.08 (11)
C1—C2—H2	119.7	C18—C19—H19	119.0
O2—C3—C2	124.61 (11)	C20—C19—H19	119.0
O2—C3—C4	113.45 (10)	C22—C20—C19	118.70 (11)
C2—C3—C4	121.95 (11)	C22—C20—C15	121.32 (11)
C5—C4—C3	119.04 (11)	C19—C20—C15	119.98 (11)
C5—C4—H4	120.5	O5—C21—H21A	109.5
C3—C4—H4	120.5	O5—C21—H21B	109.5
C4—C5—C6	121.45 (11)	H21A—C21—H21B	109.5
C4—C5—H5	119.3	O5—C21—H21C	109.5
C6—C5—H5	119.3	H21A—C21—H21C	109.5
C8—C6—C5	119.15 (11)	H21B—C21—H21C	109.5
C8—C6—C1	120.63 (11)	N3—C22—C20	125.90 (12)
C5—C6—C1	120.06 (11)	N3—C22—H22	117.1
O2—C7—H7A	109.5	C20—C22—H22	117.1
O2—C7—H7B	109.5	N3—C23—C24	109.88 (10)
H7A—C7—H7B	109.5	N3—C23—H23A	109.7
O2—C7—H7C	109.5	C24—C23—H23A	109.7
H7A—C7—H7C	109.5	N3—C23—H23B	109.7
H7B—C7—H7C	109.5	C24—C23—H23B	109.7
N1—C8—C6	125.19 (11)	H23A—C23—H23B	108.2
N1—C8—H8	117.4	N4—C24—C23	113.23 (10)
C6—C8—H8	117.4	N4—C24—H24A	108.9
N1—C9—C10	110.56 (10)	C23—C24—H24A	108.9
N1—C9—H9A	109.5	N4—C24—H24B	108.9
C10—C9—H9A	109.5	C23—C24—H24B	108.9
N1—C9—H9B	109.5	H24A—C24—H24B	107.7
C10—C9—H9B	109.5	N4—C25—C26	110.72 (10)
H9A—C9—H9B	108.1	N4—C25—H25A	109.5
N2—C10—C9	111.97 (10)	C26—C25—H25A	109.5
N2—C10—H10A	109.2	N4—C25—H25B	109.5
C9—C10—H10A	109.2	C26—C25—H25B	109.5
N2—C10—H10B	109.2	H25A—C25—H25B	108.1
C9—C10—H10B	109.2	O6—C26—C25	111.69 (10)
H10A—C10—H10B	107.9	O6—C26—H26A	109.3
N2—C11—C12	111.64 (11)	C25—C26—H26A	109.3
N2—C11—H11A	109.3	O6—C26—H26B	109.3
C12—C11—H11A	109.3	C25—C26—H26B	109.3
N2—C11—H11B	109.3	H26A—C26—H26B	107.9
C12—C11—H11B	109.3	O6—C27—C28	111.39 (10)
H11A—C11—H11B	108.0	O6—C27—H27A	109.3
O3—C12—C11	111.69 (12)	C28—C27—H27A	109.3
O3—C12—H12A	109.3	O6—C27—H27B	109.3
C11—C12—H12A	109.3	C28—C27—H27B	109.3
O3—C12—H12B	109.3	H27A—C27—H27B	108.0
C11—C12—H12B	109.3	N4—C28—C27	109.79 (10)
H12A—C12—H12B	107.9	N4—C28—H28A	109.7
O3—C13—C14	111.10 (13)	C27—C28—H28A	109.7
O3—C13—H13A	109.4	N4—C28—H28B	109.7
C14—C13—H13A	109.4	C27—C28—H28B	109.7
O3—C13—H13B	109.4	H28A—C28—H28B	108.2
			
O1—C1—C2—C3	179.90 (11)	O4—C15—C16—C17	−179.19 (11)
C6—C1—C2—C3	1.07 (16)	C20—C15—C16—C17	0.26 (17)
C7—O2—C3—C2	6.74 (17)	C21—O5—C17—C16	−5.24 (18)
C7—O2—C3—C4	−172.62 (10)	C21—O5—C17—C18	175.49 (10)
C1—C2—C3—O2	−178.94 (10)	C15—C16—C17—O5	179.04 (10)
C1—C2—C3—C4	0.37 (18)	C15—C16—C17—C18	−1.75 (19)
O2—C3—C4—C5	178.02 (10)	O5—C17—C18—C19	−178.74 (10)
C2—C3—C4—C5	−1.36 (18)	C16—C17—C18—C19	1.96 (18)
C3—C4—C5—C6	0.81 (17)	C17—C18—C19—C20	−0.67 (18)
C4—C5—C6—C8	−174.64 (11)	C18—C19—C20—C22	179.23 (11)
C4—C5—C6—C1	0.66 (17)	C18—C19—C20—C15	−0.77 (18)
O1—C1—C6—C8	−5.18 (17)	O4—C15—C20—C22	0.43 (19)
C2—C1—C6—C8	173.65 (11)	C16—C15—C20—C22	−179.02 (11)
O1—C1—C6—C5	179.58 (11)	O4—C15—C20—C19	−179.57 (11)
C2—C1—C6—C5	−1.58 (16)	C16—C15—C20—C19	0.98 (17)
C9—N1—C8—C6	−172.63 (11)	C23—N3—C22—C20	−177.67 (11)
C5—C6—C8—N1	177.35 (11)	C19—C20—C22—N3	−179.06 (12)
C1—C6—C8—N1	2.07 (18)	C15—C20—C22—N3	0.94 (19)
C8—N1—C9—C10	−112.86 (13)	C22—N3—C23—C24	102.47 (14)
C11—N2—C10—C9	178.68 (11)	C28—N4—C24—C23	170.73 (10)
C14—N2—C10—C9	−59.94 (14)	C25—N4—C24—C23	−69.17 (13)
N1—C9—C10—N2	−171.02 (10)	N3—C23—C24—N4	−173.15 (10)
C10—N2—C11—C12	176.04 (11)	C24—N4—C25—C26	−178.31 (10)
C14—N2—C11—C12	53.07 (15)	C28—N4—C25—C26	−57.12 (13)
C13—O3—C12—C11	59.03 (15)	C27—O6—C26—C25	−56.66 (13)
N2—C11—C12—O3	−55.80 (16)	N4—C25—C26—O6	57.23 (14)
C12—O3—C13—C14	−61.84 (15)	C26—O6—C27—C28	58.34 (13)
C11—N2—C14—C13	−54.87 (15)	C24—N4—C28—C27	−179.29 (10)
C10—N2—C14—C13	−175.41 (11)	C25—N4—C28—C27	58.37 (13)
O3—C13—C14—N2	60.70 (16)	O6—C27—C28—N4	−60.28 (14)

### Hydrogen-bond geometry (Å, °)

**Table:**

*D*—H···*A*	*D*—H	H···*A*	*D*···*A*	*D*—H···*A*
N1—H1···O1	0.88 (1)	1.96 (1)	2.6489 (15)	133 (2)
N1—H1···O1^i^	0.88 (1)	2.32 (1)	2.9570 (18)	129 (1)
N3—H3···O4	0.89 (1)	2.02 (1)	2.6930 (15)	132 (1)
N3—H3···O4^ii^	0.89 (1)	2.29 (1)	2.9505 (18)	132 (1)

Symmetry codes: (i) −*x*+2, −*y*+1, −*z*+1; (ii) −*x*+3, −*y*, −*z*−1.
